# Vaccine Candidates against Arenavirus Infections

**DOI:** 10.3390/vaccines11030635

**Published:** 2023-03-13

**Authors:** Takeshi Saito, Rachel A. Reyna, Satoshi Taniguchi, Kirsten Littlefield, Slobodan Paessler, Junki Maruyama

**Affiliations:** 1 Department of Pathology, University of Texas Medical Branch, Galveston, TX 77555, USA; 2 Department of Microbiology & Immunology, University of Texas Medical Branch, Galveston, TX 77555, USA

**Keywords:** arenavirus, vaccine, Lassa virus, Junin virus, virus attenuation

## Abstract

The viral family *Arenaviridae* contains several members that cause severe, and often lethal, diseases in humans. Several highly pathogenic arenaviruses are classified as Risk Group 4 agents and must be handled in the highest biological containment facility, biosafety level-4 (BSL-4). Vaccines and treatments are very limited for these pathogens. The development of vaccines is crucial for the establishment of countermeasures against highly pathogenic arenavirus infections. While several vaccine candidates have been investigated, there are currently no approved vaccines for arenavirus infection except for Candid#1, a live-attenuated Junin virus vaccine only licensed in Argentina. Current platforms under investigation for use include live-attenuated vaccines, recombinant virus-based vaccines, and recombinant proteins. We summarize here the recent updates of vaccine candidates against arenavirus infections.

## 1. Introduction

Mammarenaviruses belong to the family *Arenaviridae* and the genus *Mammarenavirus*. According to their geographic distribution and phylogenetic relationships, mammarenaviruses are further divided into Old World (OW) and New World (NW) arenaviruses [1,2,3]. Several of these cause infections in humans, ranging from asymptomatic to fatal outcomes. Lassa virus (LASV) is an OW arenavirus and the causative agent of Lassa fever (LF), a fatal hemorrhagic fever. Outbreaks of LF are reported annually in endemic western African countries, with high mortality in symptomatic patients [3,4]. Junin virus (JUNV) is a NW arenavirus and causes Argentine hemorrhagic fever (AHF). AHF is also a lethal hemorrhagic fever with severe public health consequences. Seven other mammarenaviruses, including lymphocytic choriomeningitis virus (LCMV), Lujo virus (LUJV), Machupo virus (MACV), Guanarito virus (GTOV), Sabia virus (SABV), Chapare virus (CHAPV), and Whitewater Arroyo virus (WWAV) have been reported to cause infectious diseases in humans, although the numbers of reported cases are less than both LF and AHF [1,2,5]. Due to their highly infectious nature, risk of imported cases, and use in bioterrorism, the diseases caused by mammarenaviruses are some of the most severe public health threats. There is a concern that the endemic areas of these viruses may expand due to climate change and human economic activities [6]. Establishment of countermeasures are urgently needed to overcome these threats to public health. Vaccination is one of the primary methods to prevent infectious diseases. Even though there has been much progress recently in the development of vaccines against arenaviruses, only the live-attenuated JUNV vaccine, Candid#1 [7,8,9], is approved for use in AHF-endemic countries. There are currently no FDA-approved vaccines against any arenavirus infections. In this review, the development of vaccines against arenavirus infections will be described, and the mechanism of protection of each proposed vaccine candidate will be presented.

## 2. Classification and Distribution of Arenaviruses

**Figure 1:**
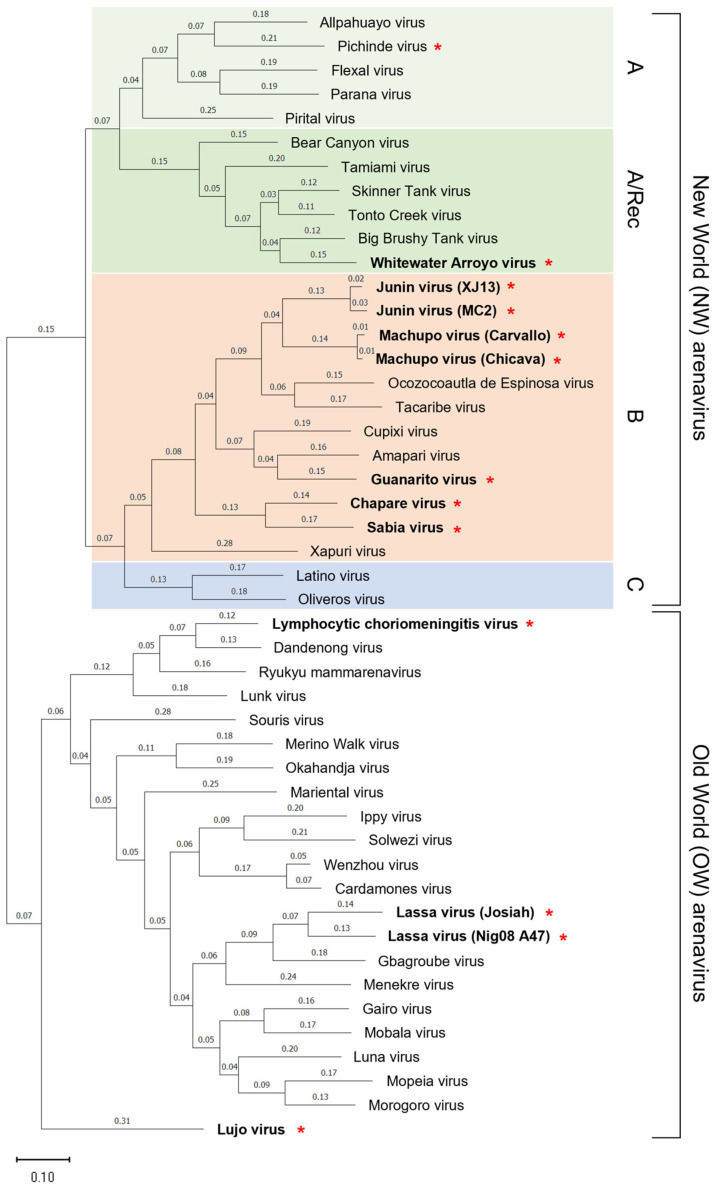
Phylogenetic relationships and classification of arenaviruses. The New World (NW) arenaviruses are subdivided into clade A (light green), clade A/Rec (green), clade B (orange), and clade C (blue). The phylogenetic tree is based on a nucleotide comparison of the NP genes. Red stars following virus names indicates the ability to infect humans. The virus names in boldface indicate Lymphocytic choriomeningitis virus and those that cause fatal hemorrhagic fevers in humans. The phylogenetic tree was drawn using MEGA11: Molecular Evolutionary Genetics Analysis version 1 [24]. The scale bar indicates substitutions per site. Accession numbers for reference sequences are: NC_010253.1 (Allpahuayo virus), NC_006447.1 (Pichinde virus), NC_010757.1 (Flexal virus), NC_010756.1 (Parana virus), NC_005894.1 (Pirital virus), NC_010256.1 (Bear Canyon virus), NC_010701.1 (Tamiami virus), EU123328.1 (Skinner Tank virus), EF619034.1 (Tonto Creek virus), EF619035.1 (Big Brushy Tank virus), NC_010700.1 (Whitewater Arroyo virus), NC_005081.1 (Junin virus, XJ13), D10072.2 (Junin virus, MC2), NC_005078.1 (Machupo virus, Carvallo), AY624355.1 (Machupo virus, Chicava), JN897398.1 (Ocozocoautla de Espinosa virus), NC_004293.1 (Tacaribe virus), NC_010254.1 (Cupixi virus), NC_010247.1 (Amapari virus), NC_005077.1 (Guanarito virus), NC_010562.1 (Chapare virus), NC_006317.1 (Sabia virus), MG976578.1 (Xapuri virus), NC_010758.1 (Latino virus), NC_010248.1 (Oliveros virus), AY847350.1 (Lymphocytic choriomeningitis virus), EU136038.1 (Dandenong virus), NC_039009.1 (Ryukyu mammarenavirus), NC_018710.1 (Lunk virus), NC_039012.1 (Souris virus), NC_023764.1 (Merino Walk virus), NC_027135.1 (Okahandja virus), NC_027134.1 (Mariental virus), NC_007905.1 (Ippy virus), NC_038367.1 (Solwezi virus), NC_026018.1 (Wenzhou virus), KC669694.1 (Cardamones virus), NC_004296.1 (Lassa virus, Josiah), GU481078.1 (Lassa virus, Nig08_A47), GU830848.1 (Gbagroube virus), GU830862.1 (Menekre virus), NC_026246.1 (Gairo virus), NC_007903.1 (Mobala virus), NC_016152.1 (Luna virus), DQ328874.1 (Mopeia virus), NC_013057.1 (Morogoro virus), and NC_012776.1 (Lujo virus).

**Figure 2:**
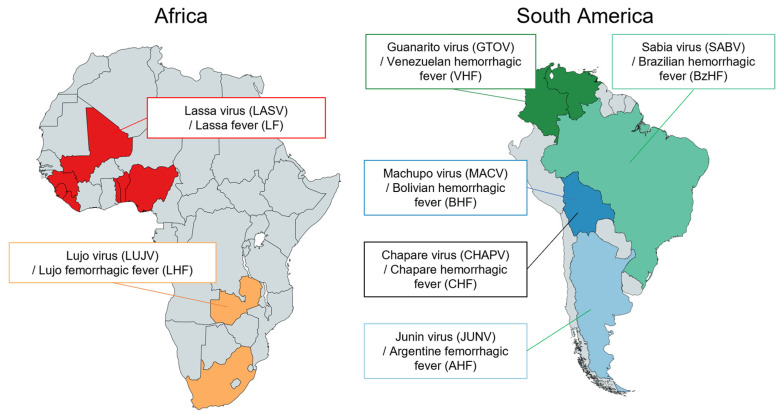
Geographic distribution of human pathogenic arenaviruses. The OW arenaviruses are mainly distributed in the African continent, with the exception of LCMV, which is found worldwide. LASV is endemic in West African countries including Guinea, Liberia, Sierra Leone, and Nigeria, and LUJV is endemic in Zambia and South Africa. The NW arenaviruses are distributed mainly in South America, with JUNV in Argentina, MACV in Bolivia, GTOV in Venezuela and Colombia, CHAPV in Bolivia, and SABV in Brazil. WWAV is found in North America.

The family *Arenaviridae* is divided into four different genera: *Antennavirus*, *Hartmanivirus*, *Reptarenavirus*, and *Mammarenavirus*. According to the latest International Committee for the Taxonomy of Viruses (ICTV) report, the genus *Mammarenavirus* currently includes 42 virus species. The *Mammarenavirus* species are further classified into OW and NW arenaviruses based on their phylogenetic relationships and their geographic distribution (Figure 1) [1,2,10,11]. OW arenaviruses are distributed predominantly on the African continent, with the exception of LCMV, which has been isolated from laboratory mice (*Mus Musculus*) worldwide. LASV is the etiological agent of LF and is endemic in West African countries including Guinea, Liberia, Sierra Leone, and Nigeria (Figure 2), corresponding with habitats of its natural rodent host, *Mastomys natalensis* [3]. Mopeia virus (MOPV) is also found in this rodent throughout Mozambique and Zimbabwe, but has not been shown to cause human disease [12,13,14]. LUJV caused a small outbreak of Lujo hemorrhagic fever (LHF) in 2008 in Zambia and South Africa (Figure 2); the natural host of LUJV is still unknown [15,16,17]. The NW arenaviruses are distributed throughout the South and North American continents and are further divided phylogenetically into four clades: Clade A, B, C, and A/Rec (D) (Figure 1 and Figure 2). The Clade B group includes human pathogenic viruses JUNV, MACV, GTOV, SABV, and CHAPV, as well as the Tacaribe virus (TCRV), which is isolated from Artibeus bats and is not known to cause human disease [11,18,19]. Hemorrhagic fevers caused by JUNV, MACV, GTOV, SABV, and CHAPV are known as Argentine Hemorrhagic Fever (AHF), Bolivian Hemorrhagic Fever (BHF), Venezuelan Hemorrhagic Fever (VHF), Brazilian Hemorrhagic Fever (BzHF), and Chapare Hemorrhagic Fever (CHF) respectively, referring to the areas where they occur. LASV, LUJV, JUNV, MACV, GTOV, SABV, and CHAPV are identified as Risk Group 4 pathogens by the World Health Organization (WHO), since they cause severe hemorrhagic diseases and have no approved vaccines and limited therapeutics. NW arenaviruses include other non-pathogenic viruses, such as Pichinde virus (PICV) belonging to the Clade A group and human-pathogenic WWAV belonging to the Clade A/Rec (D). Recently, CHF cases were reported in Bolivia in 2019 [20], BzHF cases caused by a novel strain of SABV were also reported in Brazil in 2020 [21], and a seasonal epidemic of VHF was documented in Colombia in 2021 (Figure 2) [22]. In addition, novel mammarenaviruses have been detected in rodents worldwide [13,23], but the pathogenic potential of these viruses for humans has not been verified.

## 3. Structure of Arenavirus and Its Relevance to Vaccine Development

**Figure 3:**
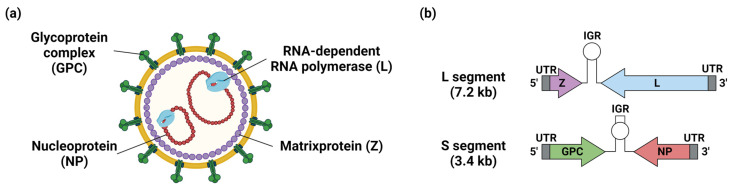
Structure of arenavirus virion and genome. (**a**) Structure of arenavirus virion showing surface glycoprotein complex (GPC,), nucleoprotein (NP), Zinc finger matrix protein (Z), and RNA-dependent RNA polymerase (L). (**b**) The genome of arenaviruses is bi-segmented, single-stranded, negative-sense RNA. The segmented genomes consist of small- (S) and large (L)-segments flanked by 5′ untranslated regions (UTRs) and 3′ UTRs. The L segment encodes Z and L, and the S segment encodes GPC and NP. The S- and L-segment encode their respective proteins using an ambisense encoding strategy, with the coding regions separated by the noncoding intergenic regions (IGRs). Figure created with BioRender (https://app.biorender.com, (accessed on 9 March 2023)).

**Figure 4:**
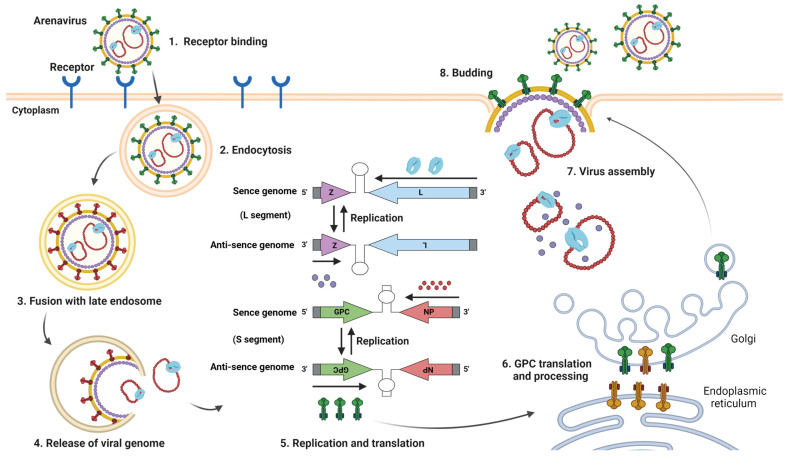
Life cycle of arenavirus. Virus entry into cells is initiated by binding GP1 to cellular receptors (1). Following attachment to the cell surface, viruses are mainly internalized by endocytosis (2). Conformational change of GPC triggered by acidic condition in the late endosomes promotes fusion between the virus and endosome membrane, leading to the release of viral genomes and replication complexes into cytosols (3 and 4). Replication complexes are released into the cytoplasm and initiate replication, transcription, and translation of the viral genome with NP and L. The transcription of viral genes begins at the 3′ ends of sense genomic vRNA and complementary anti-sense vRNA. NP and L coding regions are transcribed directly from the vRNA. GPC and Z are translated from anti-sense vRNA. A secondary stem-loop structure within IGRs of the S and L segments is responsible for transcription termination. The anti-sense vRNA serves as a template for newly synthesized vRNA (5). GPC is translated in the endoplasmic reticulum and undergoes N-linked glycosylation and cleavage of SSP by cellular signal peptidase (SPase). GPC is further cleaved into GP1 and GP2 by Subtilisin Kexin Isozyme 1/Site 1 Protease (SKI-1/S1P), and finally matured into SSP, GP1, and GP2 in the trans-Golgi network (6). Z protein utilizes the ESCRT pathway to drive transportation and assembly of viral components such as NP, L, and replication complexes at the plasma membrane. Z protein also interacts with GPC, mediating the incorporation of viral RNP complexes into GPC containing particles, leading to the release of the progeny virus from infected cells (7 and 8). Figure created with BioRender (https://app.biorender.com, (accessed on 9 March 2023)).

Mammarenaviruses are enveloped viruses with bi-segmented, single-stranded, negative-sense RNA as their viral genome (Figure 3 and Figure 4) [2,3]. The segmented genome consists of small (S) and large (L) segments. The S-segment encodes the nucleoprotein (NP) and glycoprotein precursor (GPC). GPC is cleaved during post-translational modification into a stable signal peptide (SSP), GP1, and GP2 to form a trimer on the viral membrane [2]. The L-segment encodes multi-functional matrix protein (Z) and RNA-dependent RNA polymerase (L). NP and L are required for viral genome synthesis and transcription. The S- and L-segment encode their respective proteins using an ambisense encoding strategy, with the coding regions separated by noncoding intergenic regions (IGRs). Virus entry into cells is initiated by binding GP1 to cellular receptors. LASV, LCMV, and the Clade C NW arenaviruses utilized α-dystroglycan as their cellular receptors [25]. The cellular surface receptor of LUJV is neuropilin-2, and the Clade A, B, and A/Rec NW arenaviruses mainly use transferrin receptor 1 as their receptor [25]. The T-cell immunoglobulin and mucin receptor (TIM) family, phosphatidylserine-binding receptors of the Tyro3/Axl/Mer (TAM), C-type lectins, and voltage-gated calcium channel (VGCC) subunits are also involved in viral entry into cells [25]. Following attachment to the cell surface, viruses are internalized mainly by endocytosis. Conformational changes of GPC are triggered by acidic conditions in late endosomes, promoting fusion between the viral envelope and the endosomal membrane through the function of GP2. The result is release of viral genomes and replication complexes into the cytosol. During membrane fusion, LASV and LUJV need to switch receptors to the endosomal receptors lysosomal-associated membrane protein (LAMP1) and CD63, respectively [25]. Replication complexes composed of NP and L are released into the cytoplasm where they initiate replication, transcription, and the translation of the viral genome. Transcription of viral genes begins at the 3′ ends of viral RNA (sense genomic RNA; vRNA) and complementary RNA (anti-sense genomic RNA). A secondary stem-loop structure within IGRs of the S and L segments is responsible for transcription termination. NP and L coding regions are located on the 3′ ends of the vRNA, and mRNAs encoding these viral proteins are transcribed directly from the vRNA. Thus, NP and L, as products of early infection, further promote replication and translation of the viral genome. GPC and Z are transcribed from anti-sense vRNA. The anti-sense vRNA serves as a template for newly synthesized vRNA. Z protein negatively regulates the replication, transcription, and translation processes through interaction with L and therefore controls the expression of viral proteins [26,27,28]. GPC is translated in the endoplasmic reticulum and undergoes N-linked glycosylation and cleavage of SSP by cellular signal peptidase (SPase). GPC is further cleaved into GP1 and GP2 by Subtilisin Kexin Isozyme 1/Site 1 Protease (SKI-1/S1P), and finally matures into SSP, GP1, and GP2 in the trans-Golgi network. Z protein utilizes the host endosomal sorting complex required for the transport (ESCRT) pathway to drive the transportation and assembly of viral components such as NP, L, and replication complexes at the plasma membrane. Z protein also interacts with GPC, mediating the incorporation of viral RNP complexes into GPC containing particles and leading to the release of progeny virus from infected cells [29].

Arenaviruses promote viral life cycle efficiency in host cells by disturbing the host immune response. There are well-summarized articles on the host immune suppression by arenavirus NP and Z proteins [30,31]. NPs interact with retinoic acid-inducible gene I product (RIG-I), melanoma differentiation-associated (MDA5), serine/threonine kinases IκB kinase ε (IKKε), and other factors, leading to the suppression of the type-I interferon (IFN) response as well as control of the host protein translation. LCMV, LASV, MOPV, TCRV, and PICV NPs have exonuclease (ExoN) activities which also contribute to decreased type-I IFN production by degrading dsRNA in infected cells. The ExoN activity of NPs is associated with the DEDDh (Asp-Glu-Asp-Asp) motif in the C-terminal domain. Indeed, recombinant LASV, MOPV or PICV with mutations in the active site of the ExoN domain cause higher IFN-I responses compared to wild-type recombinant viruses. It should be noted that such recombinant viruses with mutations in the ExoN domain are investigated as vaccine candidates [32,33,34]. JUNV NP has also been revealed to contain the DEDDh motif; however, it is considered incomplete without ExoN activity. The Z protein also serves as an inhibitor of immune response suppressing IFN-β production by binding RIG-I and MDA5. The surface GPC trimer is a target of neutralizing antibodies and T-cell immunity induced by viral infection or vaccination [35,36]. LASV GPC-specific non-neutralizing antibodies have also been suggested to contribute to protection from LF [37]. Since the induction of humoral and cell-mediated immunity is critical for effective vaccines, GPC has been engineered in various approaches as a pivotal element in vaccine development, as described below. NP is also thought to induce T-cell immunity similarly to GPC, and cross-reactive vaccine candidates based on epitope prediction have been investigated [38]. Understanding the sophisticated survival strategies of both viruses and host immune responses is an important factor that can be applied in vaccine development.

## 4. Immune Induction by Arenavirus Infection

Different interactions of pathogenic OW and NW arenaviruses with host immune systems have been suggested, which may affect their pathogenicity. Severe cases of LF are associated with high levels of viremia with severe immunosuppression characterized by weak or delayed type-I IFN and inflammatory cytokine responses in the early stages [39]. This immunosuppression results from LASV efficiently infecting macrophages and dendritic cells in early stages of infection without stimulating type -I IFN and cytokine responses. Thus, T-cell activation, which should follow macrophage and dendritic cell activation, does not occur, resulting in a delayed cellular immune response to LASV infection [39]. LF survivors show virus-specific CD4 and CD8 T-cell responses during the acute phase of infection, which are associated with virus clearance and recovery. Furthermore, the survival of LASV-infected cynomolgus monkeys is related to early type-I IFN response and increased CD4 and CD8 T-cells, indicating that an appropriate early immune response and induction of T-cell immunity are important for survival from LASV infection [40]. In contrast to LASV, MOPV infection induces a strong IFN and cytokine response in macrophages and dendritic cells and induces T-cell activation. As for humoral immune responses to LASV infection, even LF survivors do not produce neutralizing antibodies until several months after the acute phase. Therefore, neutralizing antibodies induced by LASV infection are unlikely to be important for virus clearance. Because of these immunological characteristics of LF, development of LF vaccine candidates has focused on the induction of T-cell immunity as well as humoral immunity.

If we focus on the induction of T-cellular immunity by vaccination, we should also evaluate its effect on hearing loss as a sequela of LF. Hearing loss affects about one-third of LF survivors, and is estimated to be permanent in about two-thirds of cases [41,42]. This sequela is a severe problem that impairs the quality of life of LF survivors. Recently, STAT1^−/−^ mice infected with LASV have been successfully used as an animal model of hearing loss [43,44,45]. This model supports the mechanism that hearing loss is due to immune-mediated injury by T-cell responses [43].

Several pathogenic NW arenavirus infections are associated with elevated type-I IFN and inflammatory cytokine levels. For example, the severity and outcome of AHF correlate with type-I IFN and cytokine levels [39]. In severe cases of AHF, robust type-I IFN and inflammatory cytokine responses occur, while these responses are not as strong in orthotopic cases. In addition, for humoral immunity, patients with AHF produce neutralizing antibodies against JUNV during the acute phase of the disease [39], which is essential for viral clearance. Furthermore, both AHF and BHF can be successfully treated with immune sera derived from survivors in the convalescent phase. Therefore, the induction of neutralizing antibodies is important for NW arenavirus vaccine development. It should be noted that immunization with Candid#1 induces a strong and protective neutralizing antibody response. The details of immune responses induced by LHF, VHF, BzHF, or CHF are under investigation. For vaccine development, a comprehensive understanding of the immune induction capacity and pathogenesis for each infectious disease is necessary for success.

## 5. Arenavirus Vaccine Development

Despite the public health threat of mammarenavirus infection, there are no FDA-approved vaccines except for JUNV Candid #1, which has only been approved for limited use in Argentina. While LF and AHF are the two major mammarenavirus infectious diseases and several vaccine candidates against them have been developed, the WHO and the Coalition for Epidemic Preparedness Innovations (CEPI) have prioritized LASV vaccine development [46,47]. The vaccine platforms and strategies outlined in this review will mainly focus on LF and AHF. Incidents of other mammarenavirus infections are rare compared to LF and AHF, although the development of vaccines against them remains crucial, as cases of VHF, BzHF, and CHF were reported recently [20,48,49]. Various vaccine platforms have been investigated, ranging from live-attenuated vaccines, artificially modified recombinant viruses, the use of other viruses as vectors, as well as proteins, DNA, and mRNA [3,4,5,7,11].

Several vaccine candidates against LF have been developed [46,47]. Three vaccine candidates, the recombinant vesicular stomatitis virus (VSV) expressing LASV GPC (rVSV∆G-LASV-GPC) [50,51,52], the recombinant measles virus expressing LASV GPC and NP (MV-LASV) [34,53], and a DNA vaccine encoding the LASV GPC gene (INO-4500) [54] have been evaluated for their safety and efficacy in phase 1 clinical trials (IAVI C102 ClinicalTrials.gov: NCT04794218, V182-001 ClinicalTrials.gov: NCT04055454, and LSV-001 ClinicalTrials.gov: NCT03805984, respectively). The results of these studies are not yet available. In comparison to LF, other arenavirus diseases have lagged in vaccine development. Even the vaccine for AHF, Candid#1, has not been approved by the FDA due to concerns about the risks of residual virulence and reversion to pathogenicity [55,56,57]. Given the recent cases of other severe arenavirus infections, such as the cases of CHF in Bolivia in 2019, BzHF in Brazil in 2020, and the seasonal epidemic of VHF in Colombia in 2021 [20,21,22] and the isolation of novel arenavirus species potentially harmful to humans [13,23], vaccines against such diseases should be developed.

Unfortunately, there are numerous obstacles that must be overcome for successful vaccine development. All highly pathogenic arenaviruses are classified as Risk Group 4 agents, and must be handled in the highest biological containment facility, biosafety level-4 (BSL-4), due to their lethality and lack of vaccines and therapeutics. The limited number of institutions with access to BSL-4 facilities further hampers vaccine development studies. Additionally, limitations of appropriate animal models for some arenavirus infections also make the investigation of vaccine candidates difficult. This review will describe the status of vaccine development for arenavirus infections as well as various bottlenecks.

### 5.1. Vaccine Candidates for LF

The development of a LF vaccine has progressed considerably in recent years [3,4,58]. Several vaccine candidates, such as replication-incompetent virus vectors, inactivated LASV, virus-like particles, and DNA vaccines, have demonstrated some effectiveness (Table 1) [4,58]. Among these, some candidates using replication-competent virus vectors have succeeded with high protective efficacy and immunogenicity, and are in further development as remarkable vaccine candidates [34,50,51,52,53].

Vaccines using replication-competent viral vectors are unique in their ability to maintain a long-term immune response. VSV, a member of the family *Rhabdoviridae*, is a non-segmented single stranded negative sense RNA virus. Recombinant VSV (rVSV) has been developed as a vaccine platform for several infectious diseases [51,59]. A vaccine approved by the FDA in 2019 for Ebola virus disease (EVD) was generated by replacing the VSV glycoprotein (G) gene with the Ebola virus glycoprotein gene (rVSV-EBOV) [51,60]. rVSV∆G-LASV-GPC was generated by replacing the G gene of VSV with the GPC gene of LASV. This vaccine candidate does not cause disease but induces a protective immune response, such as a T-cell response and neutralizing antibodies, against LASV infection in non-human primates (NHPs) and guinea pigs [50,51,52,61].

ChAdOx1-Lassa-GPC is a chimpanzee adenovirus-vectored vaccine candidate incorporating the LASV GPC gene [62]. This vaccine candidate induces both T-cell and antibody responses in vaccinated mice. A single administration of ChAdOx1-Lassa-GPC protects Hartley guinea pigs from morbidity and mortality after LASV infection [62].

A recombinant measles virus (Schwarz strain) vaccine candidate expressing both the LASV GPC and NP (MV-LASV) induces efficient protection against homologous LASV challenge after a single administration in cynomolgus macaques [34]. This vaccine candidate induces long-term immunity and protects against heterologous LASV strain challenge in this animal model [53].

The live-attenuated yellow fever virus strain 17D (YF17D) has been successfully developed as a vaccine for yellow fever [63]. This recombinant YF17D platform has also been utilized in LF vaccine development [64,65,66]. These vaccine candidates partially protected strain 13 guinea pigs from lethal LASV infection, but all survivors presented with symptoms of LF, and viral infection was not suppressed [64,65]. Moreover, the vaccination of common marmosets failed to induce adequate immunity and did not protect them from a lethal outcome [66].

A replication-incompetent adenovirus vector-based platform, Ad5 (E1-, E2b-), has been used for LF vaccine candidates [67]. Ad5 (E1-, E2b-) has high stability, a low risk of adverse events, and induces immune responses even in the presence of pre-existing adenovirus immunity [68]. Vaccination with both Ad5 (E1-, E2b-) vectors expressing the LASV-NP and GPC has been reported to protect guinea pigs from lethal LASV challenge [67].

DNA vaccines induce an immune response by electrically inoculating plasmid DNA encoding a viral antigen into the body and expressing the antigen in an organism [69]. A DNA vaccine candidate, INO-4500, encoding the LASV GPC protected guinea pigs and NHPs from both disease development and lethal outcomes [54]. Furthermore, this vaccine candidate is already in phase I human clinical trials. In its phase I trial, vaccination with INO-4500 induced high cellular immunity, including the upregulation of LASV GPC peptide-reactive CD4 and CD8 T-cells up to 48 weeks [70].

ML29 is a reassortant virus generated by clonal selection of reassortants of MOPV and LASV. ML29 is an LF vaccine candidate with adequate and broad cross-reactivity [71,72,73,74,75]. The efficacy of ML29 as a LF vaccine candidate has also been confirmed in marmosets, macaques, guinea pigs, and CBA/J mice [73,76,77]. Furthermore, in simian immunodeficiency virus (SIV)-infected macaques, the administration of ML29 did not increase clinical signs of arenavirus infection or shorten lifespan, and only a slight viremia of ML29 was observed [71]. This indicates that ML29 may prove safe in regions with high rates of human immunodeficiency virus (HIV)-related immunodeficiencies. However, recombinant ML29 has been reported to cause 100% fatal infection in STAT1^−/−^ mice [78]. This study demonstrated that CD4 and CD8 T- cell responses were involved in the acute infection of ML29, and infection with ML29 itself possibly induced hearing loss.

Other vaccine candidates have been developed using vaccinia viruses, rabies viruses, alphavirus replicons, virus-like particles, and recombinant LASV proteins [37,79,80,81,82,83]. Recently, recombinant LASV in which the IGR of the L segment is replaced with that of the S segment [84], recombinant LASV with codon deoptimized GPC genes [85], and recombinant MOPV with mutations in the ExoN domain of NP and replacement of GPC with LASV GPC (MOPEVAC_LASV_) [32,33] have been reported as novel live-attenuated vaccine candidates.

Further limiting vaccine development and advancements is the genetic diversity of LASVs. The sequence analysis of LASV isolates has shown that there are at least seven different lineages of LASV [86,87,88,89,90]. This genetic diversity raises concerns about the efficacy of potential LASV vaccines, since LF vaccine candidates are typically developed utilizing the genes of the Josiah strain in lineage IV [50,51].

Hearing loss is a sequela of LF, and this sequela is a significant problem that impairs the quality of life of LF survivors [41,42]. This characteristic sequela is a concern in the development of LF vaccines, especially vaccines inducing a robust T-cell response. Because details of the mechanism of hearing loss are not yet fully understood, there is a risk that vaccination may cause hearing loss in vaccine recipients. In the development of LF vaccines, the prevention of inducing hearing loss in recipients as well as achieving high protective efficacy is crucial.

### 5.2. Vaccine Candidates for Other Arenavirus Infections

Compared to LF vaccine candidates, vaccine development for other arenavirus infections has lagged, except for JUNV Candid#1 (Table 2). The establishment of reverse genetics technology for arenaviruses has enabled the development of various recombinant vaccine candidates. Recently, recombinant arenaviruses have been created and analyzed as candidates for attenuated live vaccines.

#### 5.2.1. Vaccine Candidates for AHF

Vaccine candidates for AHF have been studied in the past, including formalin-inactivated viruses or a heterologous virus, TCRV. Among these, live-attenuated viruses have been used successfully [8]. The live-attenuated JUNV strain, Candid#1, is the vaccine against AHF, and was established through the serial passage of the lethal XJ strain of JUNV in guinea pigs, mouse brains and fetal rhesus monkey lung cells [7,8,9]. Candid#1 is fully attenuated and engenders protective immunity against lethal JUNV infection in rhesus macaques, guinea pigs, and humans [8,91]. Vaccination with Candid#1 has been shown to induce long-term immunity against JUNV infection [91]. This vaccine became available in Argentina in 1992 to at-risk populations, but has not been approved by the FDA due to concerns about residual pathogenicity and the possibility of pathogenic reversion. During serial passage, Candid#1 acquired several mutations throughout its genome, especially within the GPC gene. The amino acid substitution from phenylalanine to isoleucine at position 427 (F427I) in the transmembrane domain (TMD) of the GP2 is highly associated with attenuation. The mutation from tyrosine to alanine at position 168 (T168A) leading to the disruption of N-glycosylation motif in the GP1 is also related to attenuation mechanisms [92]. Valine at position 64 of Z has also been reported to contribute to the pathogenicity of the lethal JUNV Romero strain. Candid#1 has glycine at this position, and the recombinant JUNV Romero with the V64G mutation in Z is completely attenuated in a guinea pig model [93]. Another report has shown that an amino acid substitution from lysine to serine at position 33 (K33S) in the SSP of JUNV Candid#1 GPC suppresses a reversion mutation at 427 (I427F) in GPC [56,94]. These findings could lead to the development of a vaccine candidate that is safer and more stable than the conventional JUNV Candid #1 vaccine strain.

Other approaches including DNA vaccines, recombinant virus vector vaccines, or recombinant viral proteins have also been investigated [95]. Tri-segmented recombinant MACV (r3MACV) consisting of two S segments containing the GPC genes of MACV, GTOV, and CHAPV, and one L segment originating from MACV, have been established using reverse genetics techniques [96]. r3MACV has been shown to induce host type-I IFN responses in vitro as well as to protect 50% of Hartley guinea pigs from a simultaneous lethal JUNV challenge [96]. Although its immune induction properties and protective effect for MACV, GTOV, and CHAPV infections are still unclear, r3MACV might be a strong candidate as a pan-NW arenavirus vaccine. MOPEVAC expressing MACV GPC (MOPEVAC_MACV_) and the pentavalent MOPEVAC vaccine candidate expressing GPCs from all pathogenic South American NW arenaviruses (MOPEVAC_NEW_) induce cross-neutralizing antibodies against JUNV in cynomolgus macaques after boost immunization [34].

#### 5.2.2. Vaccine Candidates for BHF

MACV is genetically closely related to JUNV, and vaccine candidates are being developed for MACV using similar approaches as those used for JUNV. The GPC of JUNV and MACV share structural characteristics such as similarities of N-linked glycosylation motif positions and a phenylalanine at position 438 within the MACV GPC, corresponding to the phenylalanine at position 427 of JUNV GPC [97,98]. Recombinant live-attenuated MACV vaccine candidates have been inspired by the attenuation mechanisms of the JUNV Candid#1 strain. A recombinant MACV, artificially substituted with the GPC of JUNV Candid#1 (rMACV/Cd#1-GPC), is fully attenuated in IFN-αβ/γ R^−/−^ mice, which succumb to parental MACV infection. The rMACV/Cd#1-GPC is immunogenic and induces sufficient protection against subsequent lethal challenge with MACV in this mouse model [97]. N-linked glycosylation at positions 83 (N83) and 166 (N166) on the GPC are also involved in the pathogenicity of MACV [97,98]. A recombinant MACV was formulated using an F438I substitution to disrupt these N-linked glycosylation sites within the GPC (MACVΔN83/ΔN166/F438I). This recombinant virus is fully attenuated in IFN-αβ/γ R^−/−^ mice and outbred Hartley guinea pigs [98]. Notably, MACVΔN83/ΔN166/F438I has a reduced ability to propagate in neuronal cell lines, indicating a lower risk of neuropathogenicity in recipients. Immunization with this vaccine candidate protects 100% of guinea pigs from lethal infection of MACV [98].

A variant of the MACV Carvallo strain (Car91) has also been reported to not cause disease in Hartley guinea pigs [99]. The same study suggests that attenuation of MACV Car91 is possibly due to loss of a portion of the L segment IGR [99]. Immunization with MACV Car91 induces high neutralizing antibody titers, but its protective effect against lethal MACV infection remains unknown [99]. Other vaccine candidates under investigation include DNA vaccines and alphavirus replicon vectors. A DNA vaccine candidate encoding the MACV GPC gene induced neutralizing antibodies in immunized rabbits [100]. An alphavirus RNA replicon vector simultaneously expressing both the JUNV and MACV GPC has been established using a Venezuelan equine encephalitis (VEEV) TC-83 vector. This vaccine candidate is safe and immunogenic in Hartley guinea pigs, inducing complete protection against lethal JUNV or MACV infections [101]. Both MOPEVAC_MACV_ and MOPEVAC_NEW_ induce neutralizing antibodies against MACV and protect cynomolgus macaques from lethal MACV infection [34].

#### 5.2.3. Vaccine Candidates for the Other Arenavirus Infections

There are also limited reports of vaccine candidates for the more minor arenavirus infections. A DNA vaccine candidate encoding the GTOV GPC gene induced homologous neutralizing antibodies in immunized rabbits [100]. Passive immunization with a cocktail of anti-JUNV, -MACV, and -GTOV IgG antibodies from DNA-vaccinated rabbits protected 100% of Hartley guinea pigs against lethal JUNV or GTOV infection. In addition, a combination of DNA plasmids encoding the GPC of JUNV, MACV, GTOV, and Sabia virus (SABV) was used to simultaneously vaccinate rabbits, resulting in sera that neutralized all four targets [100]. The protectiveness of this DNA vaccine candidate against lethal GTOV infection has not been revealed. Further development of this vaccine is important, as it may serve as a better platform for VHF vaccine development, since DNA vaccines can be designed to specifically activate antibody and/or T-cell responses. This vaccine candidate has the advantage of convenience in design and antigen combination and retains the potential to be a pan-arenavirus vaccine candidate. However, the limitations surrounding the practical availability of equipment required to administer DNA vaccines versus other vaccine platforms must be considered. MOPEVAC_NEW_ is also reported to induce cross-neutralizing antibodies against GTOV, CHAPV, SABV and sterile protection against GTOV in cynomolgus macaques [34]. Although evaluation of its protection using JUNV, CHAPV, or SABV infected animal models is needed, MOPEVAC_NEW_ could have potential as a multivalent vaccine against pathogenic NW arenaviruses. Unfortunately, there are no vaccine development reports to date for LF.

## 6. Issues Hindering the Development of Vaccines against Other Arenavirus Infections

Several vaccine candidates against LF, AHF, and BHF have been developed, as described above. However, vaccine developments for some arenavirus infections including LHF, VHF, BzHF, and CHF are still lacking. This disparity may be due in part to their relative rarity and the fact that only a limited number of research facilities are permitted to handle these viruses due to their high pathogenicity. To overcome these limitations, studies have been conducted using alternative arenaviruses, such as LCMV, TCRV, and PICV that are less or not pathogenic to humans and are available for use in BSL-2 or 3 facilities [88,102,103,104,105].

NHPs, such as Cynomolgus and Rhesus macaques, are considered biologically close to humans and are accordingly deemed “gold standard” animal models for arenavirus infections [8,51,52,106,107,108]. However, research with NHPs is limited due to their high cost and the numerous safety considerations that must be addressed when handling them in high-containment laboratories. Instead of NHP models, rodent models have been thoroughly utilized in studies focused on viral pathogenicity and vaccine development [71,98,109,110,111]. Since there are other excellent articles on animal models of LASV and JUNV infections [55,112,113,114,115], we describe animal models used to study other arenavirus infections. Unfortunately, a lack of established animal models for SABV and CHAPV infection remains a major barrier to research with these viruses. However, animal models for MACV and GTOV have been developed [116,117,118]. Infection with the MACV Chicava strain induces 100% lethal outcomes in Hartley guinea pigs [116]. The guinea pig has also been used as an animal model for VHF [117]. Both strain 13 and Hartley guinea pigs develop lethal outcomes after GTOV infection [118]. Rhesus monkeys are not lethally infected by GTOV, and high titers of neutralizing antibodies have been detected in these animals [118]. Strain 13 guinea pigs are also reported to show lethal outcomes after LUJV infection [119]. No animal models for BzHF and CHF, caused by SABV and CHAPV, respectively, have been established to date. Similar to the other animal models for arenavirus hemorrhagic fevers, strain 13 or Hartley guinea pigs are expected to be appropriate models for the vaccine development, although no work has been published to confirm this at the time of writing. The pathogenicity of SABV and CHAPV has also not yet been evaluated using NHP models. Development of these animal models is urgently needed; a novel strain of SABV has been isolated from hemorrhagic fever patients in 2019, and furthering vaccine development is crucial.

## 7. Conclusions

Arenavirus infections are severe infectious diseases threatening both human life and public health. The establishment of preventatives, therapeutics, and vaccine candidates is essential for the improvement of public health and the avoidance of these threats. The remarkable development of LF vaccines in recent years raises hope for use of the first LF vaccine within endemic areas. However, this hope is unlikely to become reality within the next few years, as the number of LF vaccine candidates undergoing clinical trials is very limited. As for other arenaviruses other than LASV and JUNV, vaccine development has significantly lagged. Despite these viruses causing a limited number of clinical cases, the local population within endemic areas remains at risk of fatal infection. Development of vaccines for these diseases, perhaps utilizing the knowledge gained from LF and AHF vaccine development, is urgently needed to protect these endemic regions. The application of these findings may require the development of new animal models and new vaccine platforms may need to be developed. Ideal vaccines for arenavirus infection should be established to be safe, provide lifetime immunity with a single administration, as well as induce broad and highly protective immunity against pathogenic arenavirus infections. We should continue our research with a broad perspective and deep insight to achieve this goal.

## Figures and Tables

**Table 1:** Summary of vaccine candidates for OW arenavirus infections.

Viruses	Diseases	Vaccine Platforms	Name of Vaccine Candidates	Antigen	Animal Experiment	Clinical Trial	Immumne Induction	Advantage	Disadvantage
LASV	LF	Inactivated viruses							
		Inactivated recombinant Lassa-Rabies virus	LASSARAB	GPC	Guinea pigs (80% protection)	Preclinical	Protective non-neutralizing antibodies		Partial protection in guinea pigs
		Replication-incompetent virus vectors							
		Recombinant serotype 5 adenovirus (Ad5)	Ad5 (E1-, E2b-) LASV-NP,-GPC	GPC and NP	Guinea pigs (100% protection)	Preclinical	Neutralizing antibodies	High stability, low risk of adverse event, unaffected by prior immunity to adenovirus	
		DNA vaccines candidate							
		Plasmid DNA vaccine encoding LASV GPC	INO-4500	GPC	Guinea pigs (100% protection), NHPs (100% protection)	Phase I	T cell response (Induction of GPC-reactive CD4 ant CD8 Tcell)	Long shelf life, Long term immunity	Special equipment requirements
		Replication-competent virus vectors							
		Vesicular stomatitis virus vector	rVSVΔG-LASV-GPC	GPC	Guinea pigs (100% protection), NHPs (100% protection)	Phase I	Neutralizing antibodies T cell response	Long term immunity, Cross protection among LASV lineages	Possible effects of preimmunity to VSV, Mild side effects
		Vaccinia virus vector	V-LSG	GPC	Cynomolgus macaques (67% protection), Rhesus macaques (100% protection)	Preclinical	T cell response (Cell-mediated immunity)	Good safety, stable antigen expression and convenient storage	Partial protection in the animal models
		Chimpanzee adenovirus vector	ChAdOx1-Lassa-GPC	GPC	Guinea pigs (100% protection)	Preclinical	T cell response (Cell-mediated immunity)	Unaffected by prior immunity to human adenovirus	
		Measles Schwarz virus vector	MV-LASV (MeV-NP)	GPC and NP_EXON_	NHPs (100% protection)	Phase I	T cell response (Cell-mediated immunity)	Long term immunity, Cross protection among LASV lineages	
		Yellow fever virus 17D vector	YF17D/LASV-GPC	GPC	Guinea pigs (80% protection), Common marmosets (0% protection)	Preclinical	T cell response (Cell-mediated immunity)	Potential as a bivalent vaccine to YF and LF	Partial protection in the animal models Possible effects of preimmunity to YF
		Recombinant live-attenuated virus							
		Recombinant LASV with the replacement of L segment IGR with that of S segment	rLASV(IGR/S-S)	Whole virus	Guinea pigs (100% protection)	Preclinical	T cell response (Cell-mediated immunity)	Genetically stable in vitro	
		Recombinant LASV with codon deoptimized GPC genes	rLASV-GPC/CD	Whole virus	Guinea pigs (100% protection)	Preclinical	T cell response (Cell-mediated immunity)	Genetically stable in vitro	
		Recombinant MOPV with NP_ExoN_ and LASV GPC	MOPEVAC_LASV_	Whole virus	Cynomolgus macaques (100% protection)	Preclinical	T cell response (Cell-mediated immunity)	Genetically stable in vitro	
		Reasortant of LASV and MOPV							
		Reasortant of LASV and MOPV	ML29	Whole virus	Guinea pigs (100% protection), NHPs (100% protection)	Preclinical	T cell response (Cell-mediated immunity)		Lethal infection in Stat1^−/−^ mouse
LUJV	LHF	Not available							

**Table 2:** Summary of vaccine candidates for NW arenavirus infections.

Viruses	Diseases	Vaccine Platforms	Name of Vaccine Candidates	Antigen	Animal Experiment	Clinical Trial	Advantage	Disadvantage
JUNV	AHF	Live-attenuate virus						
			Candid#1	Whole virus	Guinea pigs (100% protection), Rhesus macaques (100% protection)	Avaiable in Argentina	Long term immunity	Risk of revertant to high pathogenicity
		Recombinant live-attenuated virus						
		Recombinant JUNV Candid#1 with K33S mutaion in GPC	K33S rCan	Whole virus	Guinea pigs (100% protection)	Preclinical	Reduced risk of revertant to high pathogenicity	
		Tri-segmented recombinant virus	r3MACV	Whole virus	Guinea pigs (50% protection)	Preclinical	Potential as multivalent vaccine	
		Recombinant MOPV with NP_ExoN_ and NW arenavirus GPCs	MOPEVAC_NEW_	Whole virus	No data	Preclinical	Potential as multivalent vaccine	
		DNA vaccine candidate						
		DNA vaccine	DNA vaccine	GPC	No data	Preclinical	Long shelf life	Special equipment requirements
MACV	BHF	Recombinant live-attenuated virus						
		Tri-segmented recombinant virus	r3MACV	Whole virus	No data	Preclinical	Potential as multivalent vaccine	Risk of revertant to high pathogenicity
		Recombinant live-attenuated virus	rMACV/Cd#1-GPC	Whole virus	Guinea pigs (100% protection)	Preclinical		Risk of revertant to high pathogenicity
		Recombinant live-attenuated virus	rMACVΔN83/ΔN166/F438I	Whole virus	Guinea pigs (100% protection)	Preclinical		Risk of revertant to high pathogenicity
		Recombinant live-attenuated virus	Car91	Whole virus	No data (Partially protection in Guinea pigs from GTOV)	Preclinical		Risk of revertant to high pathogenicity
		Recombinant MOPV with NP_ExoN_ and MACV GPC	MOPEVAC_MACV_	Whole virus	Cynomolgus macaques (100% protection)	Preclinical		
		Recombinant MOPV with NP_ExoN_ and NW arenavirus GPCs	MOPEVAC_NEW_	Whole virus	Cynomolgus macaques (100% protection)	Preclinical	Potential as multivalent vaccine	
		DNA vaccine candidate						
		Plasmid DNA vaccine encoding MACV GPC	DNA vaccine encoding the MACV GPC	GPC	No data	Preclinical	Long shelf life	Special equipment requirements
GTOV	VHF	DNA vaccine candidate						
		Plasmid DNA vaccine encoding GTOV GPC	DNA vaccine encoding the GTOV GPC	GPC	No data	Preclinical	Long shelf life	Special equipment requirements
		Recombinant live-attenuated virus						
		Recombinant MOPV with NP_ExoN_ and NW arenavirus GPCs	MOPEVAC_NEW_	Whole virus	Cynomolgus macaques (100% protection)	Preclinical	Potential as multivalent vaccine	
SABV	BzHF	Recombinant live-attenuated virus						
		Recombinant MOPV with NP_ExoN_ and NW arenavirus GPCs	MOPEVAC_NEW_	Whole virus	No data	Preclinical	Potential as multivalent vaccine	Need to be verified in animal experiments
CHAPV	CHF	Recombinant live-attenuated virus						
		Recombinant MOPV with NP_ExoN_ and NW arenavirus GPCs	MOPEVAC_NEW_	Whole virus	No data	Preclinical	Potential as multivalent vaccine	Need to be verified in animal experiments

## Data Availability

There are no supporting data associated with this article.
